# LINC01006 promotes cell proliferation and metastasis in pancreatic cancer via miR-2682-5p/HOXB8 axis

**DOI:** 10.1186/s12935-019-1036-2

**Published:** 2019-12-02

**Authors:** Luyang Zhang, Yunjian Wang, Ling Zhang, Guohua You, Congyu Li, Bo Meng, Minghe Zhou, Min Zhang

**Affiliations:** 1 Department of Hepatobiliary Surgery, Affiliated of Cancer Hospital of Zhengzhou University, 127 Dongming Road, Zhengzhou, 450008 Henan China; 2 Department of Ultrasonography, Affiliated of Cancer Hospital of Zhengzhou University, Zhengzhou, 450008 Henan China

**Keywords:** LINC01006, miR-2682-5p, HOXB8, PC

## Abstract

**Background:**

Pancreatic cancer (PC) is one of the deadliest cancers about the digestive system. Recent researches have validated that long non-coding RNAs (lncRNAs) play vital roles in various cancers, while the function of LINC01006 in PC is rarely clarified.

**Aim of the study:**

Investigation of the specific role of LINC01006 in PC.

**Methods:**

LINC01006 expression was examined by RT-qPCR. CCK-8, EdU, transwell, wound healing, and western blot assays were carried out to explore the function of LINC01006 in PC. The interaction among LINC01006, miR-2682-5p and HOXB8 was verified by luciferase reporter, RIP and ChIP assays.

**Results:**

The expression of LINC01006 was markedly upregulated in PC tissues and cells. Furthermore, LINC01006 knockdown inhibited PC cell proliferation, invasion and migration, and upregulation of LINC01006 led to the opposite results. Besides, miR-2682-5p expression was downregulated and negatively regulated by LINC01006 in PC. Meanwhile, LINC01006 could bind with miR-2682-5p in PC. Moreover, miR-2682-5p negatively regulated HOXB8 expression and there was a binding site between miR-2682-5p and HOXB8 in PC. Additionally, miR-2682-5p overexpression or HOXB8 knockdown rescued the promotive effects of LINC01006 upregulation on PC cell progression. Similarly, miR-2682-5p inhibition or HOXB8 overexpression countervailed the repressive role of LINC01006 downregulation in PC cell progression. In addition, the transcription factor HOXB8 could activate LINC01006 transcription in PC.

**Conclusions:**

LINC01006 promotes cell proliferation and metastasis in pancreatic cancer via miR-2682-5p/HOXB8 axis, which may facilitate the treatment for PC.

## Background

Unlike other cancers whose survival rates were improved, the incidence and mortality of pancreatic cancer (PC) both keep on increasing [1, 2]. Overall, the 5-year survival rate of PC patients is 8%. It is estimated that by 2030, PC will be second major cause of tumor-associated mortality in the US [3, 4]. Cell metastasis is one of the most distinct features of PC [5]. Chemotherapy, mostly focusing on gemcitabine and gemcitabine-centered combinations, is largely used for these unresectable PC subjects [6, 7]. Despite the fact that chemotherapy has been widely employed in the treatment of PC [8], the absence of reliable diagnostic symptoms in early stage of PC renders it difficult to treat patients at appropriate time [9]. Most of PC subjects suffered from a very dreadful prognosis, living less than 1-year due to chemoresistance [10]. Although quite a few signaling pathways have been identified to be capable of serving as biomarkers of PC treatment [11, 12], the overall prognosis is still disappointing [2]. Therefore, it is imperative to explore and develop novel biomarkers for the improvement of PC.

Long non-coding RNAs (lncRNAs) are noncoding transcripts with a length of more than 200 nucleotides [13, 14]. Numbers of previous researches have demonstrated that lncRNAs take part in malignant neoplasia. For example, lncRNA CRNDE/miR-181a-5p/Wnt/β-catenin signaling pathway promotes the occurrence of colorectal cancer [15]. LncRNA-CCAT1 inhibits miR-152 expression to enhance cell growth in intrahepatic cholangiocarcinoma [16]. LncBRM activates YAP1 signaling to promote self-renewal of stem cells in liver cancer [17]. Moreover, a variety of lncRNAs have been confirmed to affect the development of PC. For instance, lncRNA-NUTF2P3-001/miR-3923/KRAS accelerates the progression of PC [18]. LncRNA-ATB functions as a tumor suppressor in PC [19]. LncRNA HOTAIR sponges miR-613 to promote cell proliferation and metastasis in PC through targeting notch3 [20]. Despite the fact that LINC01006 has been discovered to participate in gastric cancer progression [21], its influences in PC sustain unclear.

The aim of this paper was to investigate the biological role and molecular mechanism of LINC01006 in PC, and it was verified that LINC01006 functioned as a sponge of miR-2682-5p and regulated HOXB8 expression to promote cell growth and metastasis in PC, offering a novel agent for the treatment of PC.

## Materials and methods

### Clinical specimens

30 pairs of PC and adjacent non-tumor tissue samples were resected and acquired from patients at Affiliated of Cancer Hospital of Zhengzhou University. Collected samples were freezing and stored at a temperature of − 80°C. Furthermore, all patients were informed and wrote on the consent of utilizing their tissue samples for study. Procedures of this work were approved by Ethics Committee of Affiliated of Cancer Hospital of Zhengzhou University. The use of patient samples conformed to the declaration of Helsinki.

### Microarray analysis

LncRNA expression patterns in PC specimens and matched non-cancerous tissues (n = 3 per group) were monitored applying the Affymetrix Genechip miRNA 4.0 array (KangChen Biotech, Shanghai, China). AGCC software (Affymetrix, Santa Clara, CA, USA) was for data collection and analysis. Fold change above 2 was thought as significant change.

### Cell culture

Human PC cell lines including PANC-1, AsPC-1, SW1990, BxPC-3, the pancreatic duct epithelial cell line HPDE6-C7 and human renal epithelial cell line 293T were purchased from the Shanghai Cell Bank of the Chinese Academy of Sciences (Shanghai, China), and then cells were all incubated under 37 °C in 5% CO_2_ in DMEM (Gibco, Grand Island, NY, USA) added with 10% fetal bovine serum (FBS; Gibco) and 1% penicillin‐streptomycin (Thermo Fisher Scientific, Waltham, MA, USA).

### RT-qPCR

By use of Trizol reagent (Invitrogen, Carlsbad, CA, USA), total RNA was isolated from cultured cells. Next, the first cDNA strand was synthesized and amplified through a Prime Script RT reagent kit (Promega, Madison, WI, USA). The relative expression of RNAs was determined by RT‐qPCR with the SYBR-Green Real-Time PCR Kit (Applied Biosystems, Foster City, CA, USA) on ABI 7500 real-time PCR system (Applied Biosystems). Lastly, the relative genes expression levels were detected by use of the 2^−∆∆Ct^ method. At the same time, GAPDH as an internal control gene of lncRNA and U6 gene as miRNA.

### Cell transfection

Before cell transfection, PANC-1 and BxPC-3 cells were plated in six-well plates in DMEM with 10% FBS, and then cultured for 1 day. After that, transfection was performed in PANC-1 or BxPC-3 cells by use of Lipofectamine 3000 (Invitrogen). Short hairpin RNA (shRNA) against LINC01006 (sh-LINC01006#1, sh-LINC01006#2) or HOXB8 (sh-HOXB8#1, sh-HOXB8#2) and negative control shRNA (shNC) were bought from GenePharma Corporation (Suzhou, China). The overexpressing plasmids, pcDNA3.1/LINC01006, pcDNA3.1/HOXB8 and empty pcDNA were constructed by ZonHon Biopharma Institute (Changzhou, China). MiR-2682-5p mimics and a miR-2682-5p mimic NC were constructed by GenePharma Corporation. After 2 days, transfected cells were collected, and at last RT-qPCR was adopted to test the transfection efficacy.

### CCK-8

PANC-1 or BxPC-3 cells (1 × 10^3^ cell/well) were plated in 96-well plates, and then incubated for 24 h. Cellular proliferation was used to monitor by Cell Counting Kit‐8 (CCK‐8; Dojindo, Kumamoto, Japan). Besides, 100 µl of CCK8 solution was added at 0, 24, 48, 72 or 96 h respectively. Finally, the microplate reader (Bio-Tek, Winooski, VT) was adopted to detect the absorbance of 450 nm.

### Western blot

Based on using RIPA buffer (Sigma-Aldrich, St. Louis, MO, USA), proteins were extracted from PANC-1 or BxPC-3 cells, separated through SDS-PAGE (Bio-Rad Laboratories, Hercules, CA, USA) and then transferred onto PVDF membranes (Millipore, Billerica, MA, USA). When PVDF was sealed with 5% skim milk, anti-HOXB8, anti-GAPDH antibody (Abcam, Cambridge, USA) was incubated with it overnight at 4 °C. Subsequently, secondary antibodies were cultured with it in a dark room at 37 °C for 1 h. Finally, the protein bands were visualized with the application of enhanced ECL system (Tanon, Shanghai, China). GAPDH or β-actin was used as an internal control.

### RIP assay

RIP assay was performed by use of the Magna RIP RNA‐Binding Protein Immunoprecipitation Kit (Millipore). PANC-1 or BxPC-3 cells were collected and then lysed using an RIP lysis buffer (Solarbio, Beijing, China). Next, anti-Ago2 or anti-IgG control (Abcam) was incubated with cell lysate overnight at 4 °C, and protein A magnetic beads were added into cell lysate. Coprecipitated RNA bound to primary Ago2 antibody was isolated and purified. CDNA was reverse transcribed from RNA and the relative enrichment of LINC01006 and miR-2682-5p were analyzed by RT-qPCR.

### EdU proliferation assay

Transfected PANC-1 or BxPC-3 cells were incubated in the dark with 10 μM EdU reagents for 2 h. Cells were then fixed in phosphate buffer saline (PBS; Thermo Fisher Scientific) containing 4% paraformaldehyde (PFA; Sigma-Aldrich) for 15 min. Subsequently, 2 mg/ml glycine (Solarbio, Beijing, China) and 0.5% Triton X-100 (Solarbio) were added into cells. Thereafter, DAPI (Sigma-Aldrich) was added to each well and cells were incubated in the dark for half an hour. Images were observed applying a fluorescence microscope (Nikon, Tokyo, Japan).

### Transwell assay

Transwell chamber (Costar, Boston, MA, USA) with Matrigel (BD Biosciences, San Jose, CA, USA) was used to carry out invasion assay. The upper chamber was added with transfected PANC-1 or BxPC-3 cells with serum-free medium, while the bottom chamber was added with medium containing 10% FBS. After incubating for 2 days, invasive cells were fixed with 4% PFA and stained with 0.5% crystal violet (Sigma-Aldrich). Invasive cells were then visualized and counted in five random fields via the microscope.

### Wound healing assay

Wound healing assay was carried out to measure the migration capacity of cells. Briefly, PANC-1 or BxPC-3 cells were added to 6-well plates. Cells were maintained in complete culture medium for 16 h. We then scratched the monolayer. After that, cells were incubated for 1 day in medium without FBS. Wound width was visualized and measured under the microscope.

### Subcellular fractionation

PANC-1 or BxPC-3 cells were lysed by hypotonic fractionation buffer containing protease inhibitor cocktail (Roche, Mannheim, Germany). The supernatant and pellet were separated via centrifugation at 4 °C for 10 min. After that, further centrifugation of the supernatant was performed for 1 h so as to separate the membrane fraction cytosol and the cytosol. Membrane resuspension buffer containing protease inhibitor cocktail was utilized to resuspend the pellet. U6 or GAPDH were detected as control.

### RNA pull-down assay

LINC01006 RNAs were biotin-labeled via Biotin RNA Labeling Mix (Roche). Thereafter, PANC-1 or BxPC-3 cells were treated with Biotinylated LINC01006 RNAs. Magnetic beads were then added into cells. After that, cells were subjected to washing buffer. SDS-PAGE (Bio-Rad Laboratories) was used to separate lncRNA-interacting proteins. After the gels were stained with silver, the proteins were detected by Western blot.

### Luciferase reporter assay

The wild-type or mutant sequences of miR-2682-5p in LINC01006 (LINC01006-WT or LINC01006-Mut) or HOXB8 (HOXB8-WT or HOXB8-Mut) was subcloned into the pmirGLO vector and then co-transfected with miR-2682-5p mimics or NC mimics into PANC-1 or BxPC-3 cells. Simultaneously, LINC01006 promoter-WT or its mutant (LINC01006 promoter-Mut) was co-transfected into 293T or PANC-1 cells with pcDNA3.1/HOXB8 or pcDNA3.1. Transfection was carried out by using Lipotransfectamine 3000. Dual-Luciferase Report Assay (Promega) was performed after transfection for 48 h.

### Chromatin immunoprecipitation assay

Briefly, PANC-1 or BxPC-3 cells were cross-linked with 1% PFA. After sonicating and pre-clearing, cells were incubated with appropriate antibodies. The immune complex was added with protein A/G Sepharose CL-4B beads (Thermo Fisher Scientific). Eluted DNA was acquired after washing beads and subjected to RT‐qPCR analysis.

### Animal experiments

We purchased 4-week-old female BALB/c nude mice from the Shanghai Experimental Animal Center of Chinese Academy of Sciences (Shanghai, China). With the permission from Animal Care and Experiment Committee of Affiliated of Cancer Hospital of Zhengzhou University, we carried out the animal experiments. The 1 × 10^6^ BxPC-3 cells were transfected with LINC01006 knockdown vectors or negative control (GenePharma), followed by subcutaneous innoculation into BALB/C nude mice (five mice in a group). Five mice injected with BxPC-3 cells transfected with sh-NC were used as negative control so that we can assess the tumor growth in sh-LINC01006 group. Every 4 days, we measured tumor developments. Four week later, we detected the tumor weight from the euthanized mice. The lungs metastasis nodes on the lung surface were calculated.

### Immumohistochemical staining

Tissue samples from nude mice were preserved and treated, as documented previously [22]. Primary antibodies against Ki-67 (Abcam) and PCNA (Abcam) were utilized.

### Statistical analysis

Firstly, statistical analysis was performed through SPSS 21.0 software (IBM Corporation, Armonk, NY, USA). Next, results were presented as mean ± SD. At the same time, one-way ANOVA or Student’s t-test was applied to reveal the statistical significance. The expression correlation among LINC01006, HOXB8 and miR-2682-5p was analyzed via Pearson correlation analysis. Last but not least, each experiment was performed in triplicate and P <  0.05 was deemed statistically significant.

## Results

### LINC01006 is highly expressed in PC and promotes the proliferation and metastasis of PC cells

**Fig. 1:**
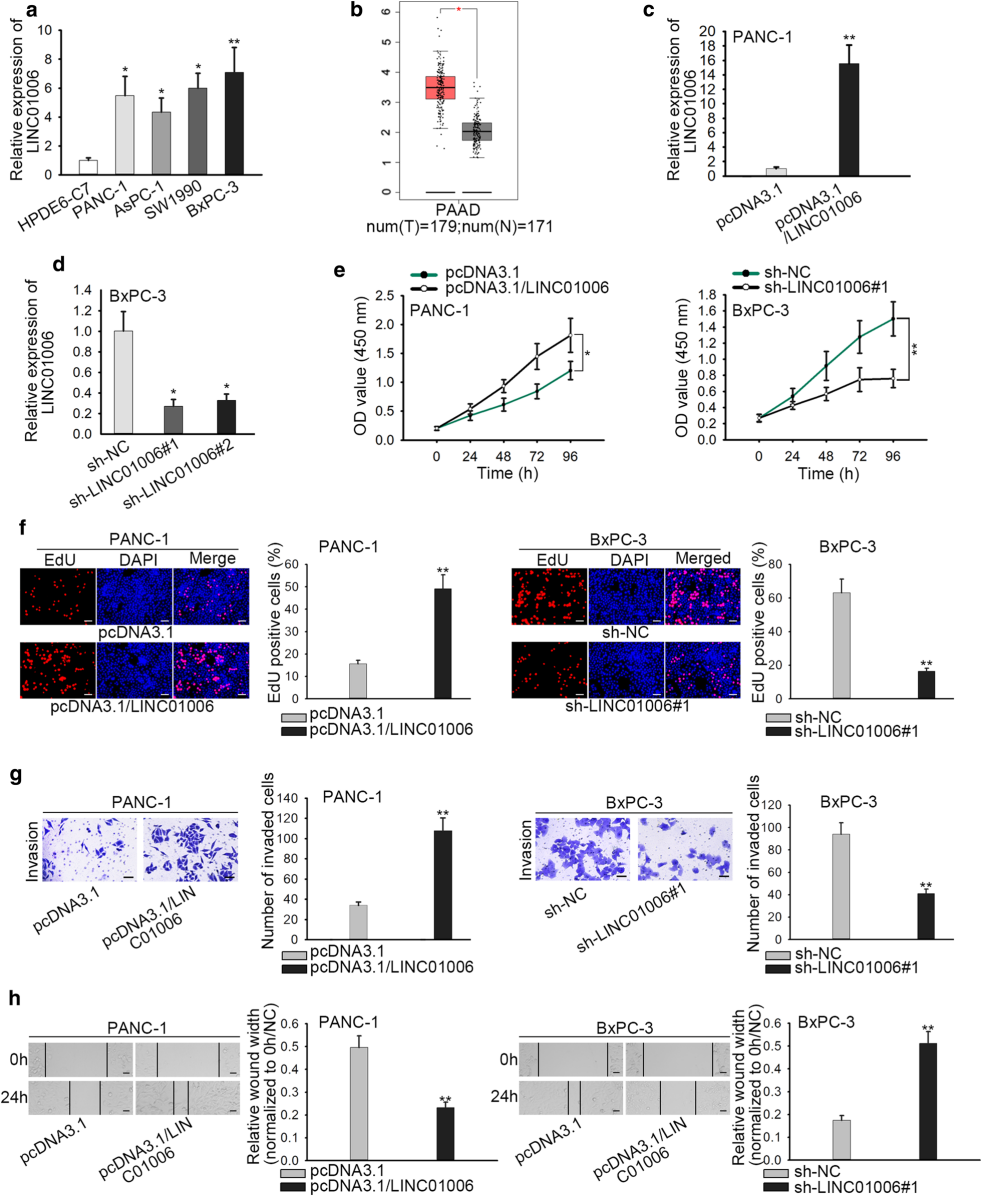
LINC01006 is highly expressed in PC and promotes the proliferation and metastasis of PC cells. **a** RT-qPCR assay was used to detect the expression of LINC01006 in PC cell lines and normal human pancreatic duct epithelial cell line. **b** According to the TCGA database, LINC01006 expression was upregulated in PC tissues. **c**, **d** The expression of LINC01006 in transfected cells was measured by RT-qPCR assay. **e**, **f** CCK-8 and EdU assays were performed to examine the proliferation ability of transfected cells. **g** Transwell assay was exercised to cell invasion ability in transfected cells. **h** Wound healing assay was employed to test cell migration ability in transfected cells. *P < 0.05, **P < 0.01

A previous lncRNA-seq was conducted to reveal the differentially expressed lncRNA in PC tissues compared with normal ones. Top ten overexpressed lncRNAs were exhibited, among which six lncRNAs have been well-identified in PC previously (Additional file 1: Fig. S1A). Regarding the rest of lncRNA, we have compared their expression pattern within normal human pancreatic duct epithelial cell line (HPDE6-C7) and PC cell lines. It indicated the specific overexpression of LINC01006 in PC cell lines, particularly in PANC-1 and BxPC-3 cells (Fig. 1a) and no significantly altered expression was observed in LINC00641, LINC00265 and LOXL1-AS1 (Additional file 1: Fig. S1B). Furthermore, the results from TCGA database showed that LINC01006 was dramatically upregulated in PC in comparison with adjacent normal tissues (Fig. 1b). To validate the function of LINC01006 in PC cellular behaviors, LINC01006 overexpression or knockdown vector was transfected into cells to increase or decrease the expression of LINC01006 (Fig. 1c, d). CCK-8 and colony formation assay manifested that cell proliferation ability in PANC-1 and BxPC-3 cells was enhanced by LINC01006 overexpression and weakened by LINC01006 silence (Fig. 1e, f). Transwell assay showed that upregulated LINC01006 promoted cell invasion and downregulation of it yielded the opposite effect on cell invasion in PANC-1 and BxPC-3 cells (Fig. 1g). Wound healing assay revealed that cell migration was accelerated after cells were transfected with pcDNA3.1/LINC01006 whereas suppressed in cells transfected with sh-LINC01006#1 (Fig. 1h). Moreover, to further confirm the biological function of LINC01006 in vivo, we have inoculated LINC01006-knockdown (sh-LINC01006) or control (sh-NC) cells into nude mice to establish the xenografts. As observed in Additional file 2: Fig. S2A, tumors developed from sh-LINC01006 cells were smaller than that from sh-NC cells. The growth rate of tumors derived from LINC01006-knockdown cells was inhibited compared with that in sh-NC group (Additional file 2: Fig. S2B). Meanwhile, tumors in sh-LINC01006 group were lighter than those in negative control group (five mice) (Additional file 2: Fig. S2C). Immumohistochemical staining demonstrated that the positive rate of Ki-67 and PCNA was reduced by LINC01006 knockdown (Additional file 2: Fig. S2D). Regarding metastasis property, immumohistochemical staining depicted that the lung metastasis nodes were decreased by LINC01006 depletion, in comparison to sh-NC group (Additional file 2: Fig. S2E). Taken together, LINC01006 is highly expressed and promotes cell proliferation and metastasis in PC.

### LINC01006 serves as a sponge of miR-2682-5p in PC

**Fig. 2:**
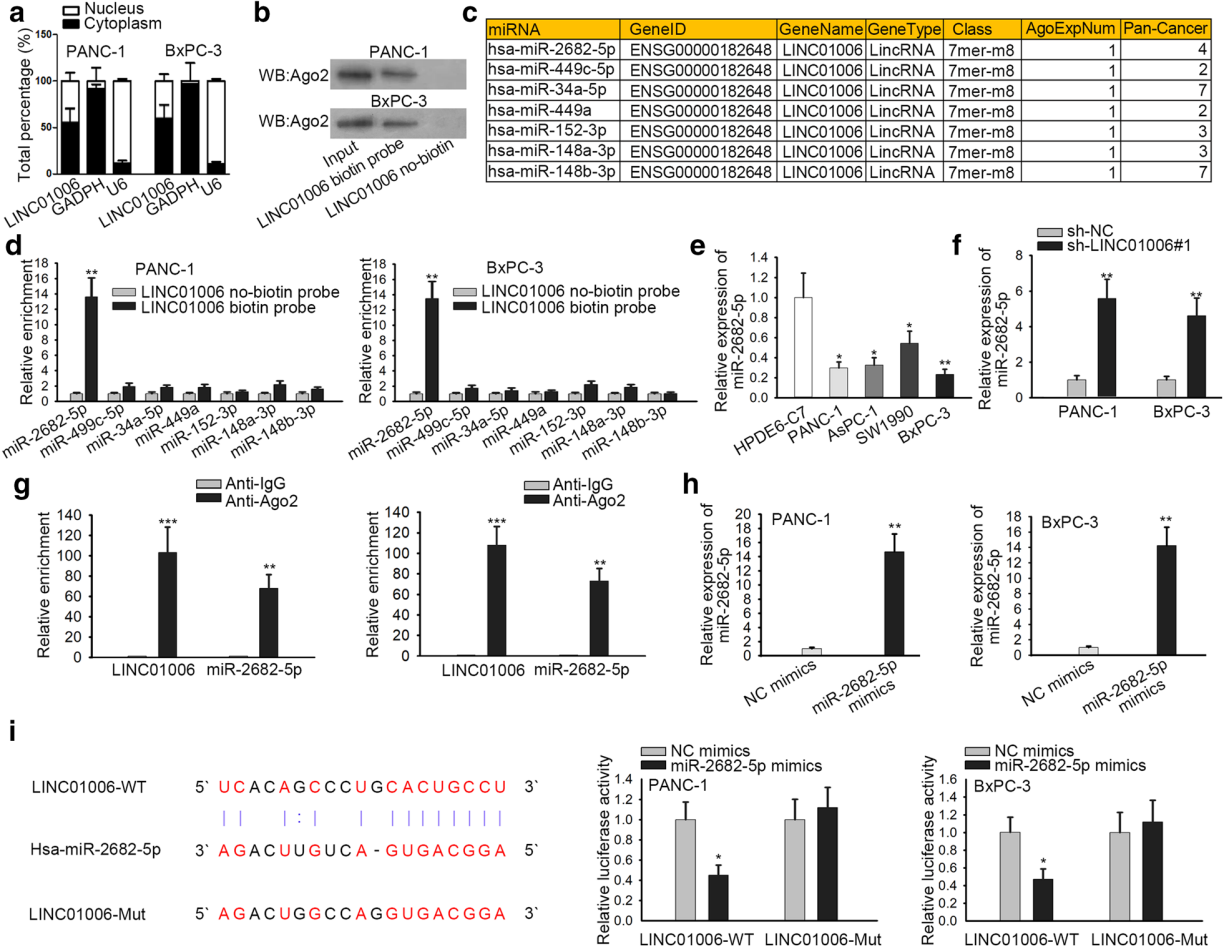
LINC01006 serves as a sponge of miR-2682-5p in PC. **a** Subcellular fractionation was utilized to detect the distribution of LINC01006 in nuclear and cytoplasm. **b** Western blot assay was employed to confirm the combination between LINC01006 and Ago2. **c** MiRNAs that could bind with LINC01006 were obtained by referring to starbase. **d** The binding ability among LINC01006 and miRNAs was examined by RNA pull down assay. **e** The expression of miR-2682-5p in PC cell lines and normal human pancreatic duct epithelial cell line was detected by RT-qPCR. **f** RT-qPCR assays were used to detect the expression of miR-2682-5p in transfected cells. **g** RIP assay was carried out to verify that LINC01006 could bind with miR-2682-5p. **h** RT-qPCR assay analyzed the overexpression efficiency of miR-2682-5p in PANC-1 and BxPC-3 cells. **i** The binding site between LINC01006 and miR-2682-5p was obtained from starBase, which was then verified by luciferase reporter assay. *P < 0.05, **P < 0.01, ***P < 0.001

Next, we have researched the regulatory mechanism of LINC01006 in PC. Inspired by the well-recognized ceRNA hypothesis [23] in RNA induced silencing complex (RISC), we detected the localization of LINC01006 in PC cells and discovered that it mainly located in the cytoplasm (Fig. 2a). it was confirmed that LINC01006 could combine with Ago2 protein, a crucial part of RISC (Fig. 2b). According to starBase (http://starbase.sysu.edu.cn/), seven candidate genes were screened out to potentially interact with LINC01006 (Fig. 2c). Then, we identified miR-2682-5p as a potential target gene of LINC01006 through RNA pull-down analysis because biotin labeled LINC01006 could pull down miR-2682-5p not the rest six miRNAs (Fig. 2d). We also found that miR-2682-5p expression was lowly expressed in PC cell lines, indicating its potential involvement in PC. Afterwards, whether LINC01006 could regulate miR-2682-5p was probed. Results suggested miR-2682-5p was considerably upregulated by LINC01006 silence in PANC-1 and BxPC-3 cells (Fig. 2f). RIP assay verified that LINC01006 and miR-2682-5p were precipitated by Anti-Ago2, indicating the co-expression of LINC01006 and miR-2682-5p in RISC (Fig. 2g). The level of miR-2682-5p was significantly increased using miR-2682-5p mimics for the preparation of luciferase reporter assay (Fig. 2h). As shown in Fig. 2i, miR-2682-5p had binding sites for LINC01006, and luciferase reporter assay indicated that miR-2682-5p mimics decreased the activity of LINC01006-WT reporter rather than that of LINC01006-Mut reporter. These data display that LINC01006 serves as a sponge of miR-2682-5p in PC.

### HOXB8 is a target gene of miR-2682-5p in PC

**Fig. 3:**
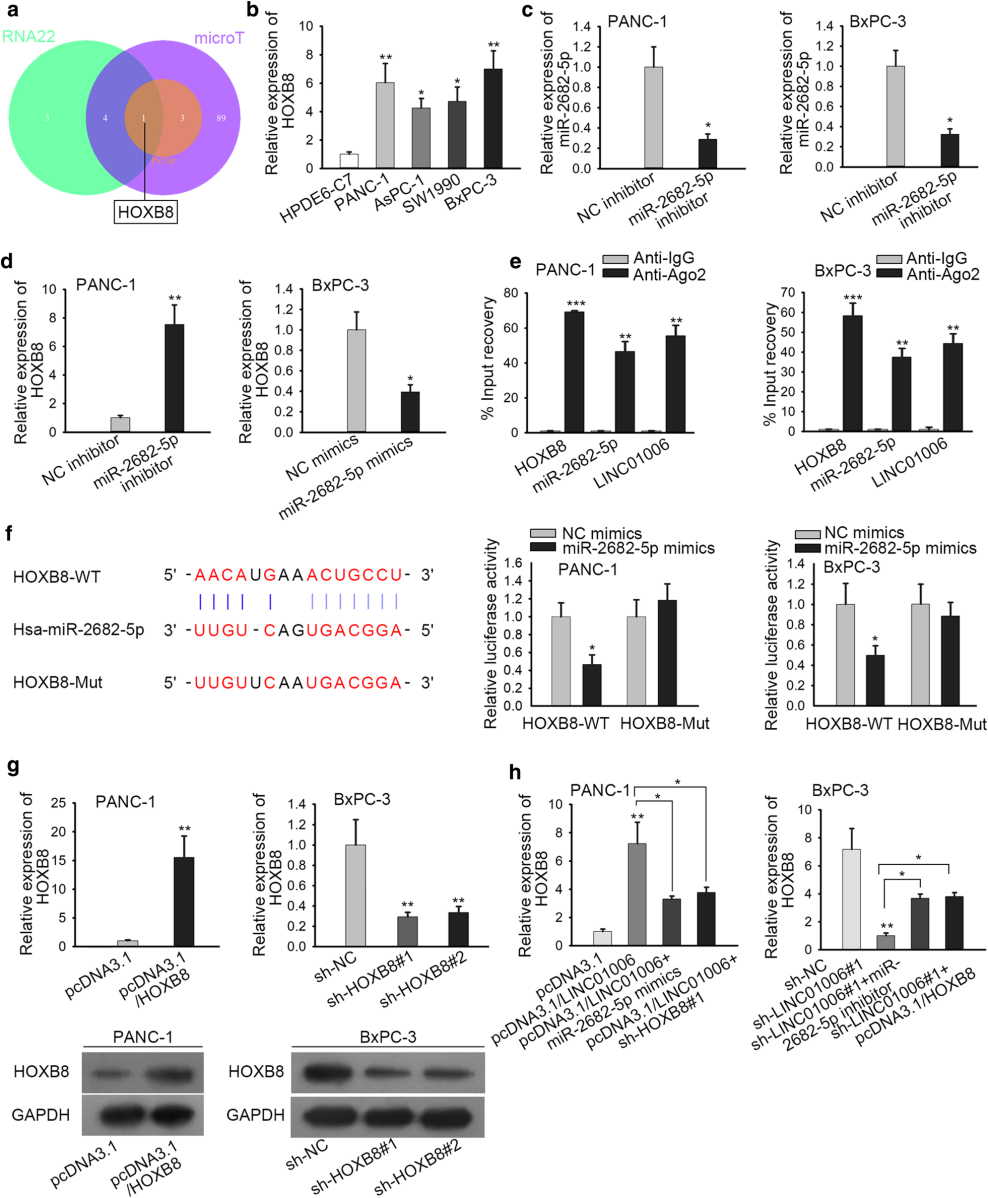
HOXB8 is a target gene of miR-2682-5p in PC. **a** The Venn diagram displayed the potential target gene of miR-2682-5p by utilizing RNA22, microT and PicTar databases. **b** The expression of HOXB8 in PC cell lines and normal human pancreatic duct epithelial cell line was detected by RT-qPCR. **c**, **d** RT-qPCR analysis was used to detect the expression of miR-2682-5p and HOXB8 in transfected cells. **e** The binding relation among LINC01006, miR-2682-5p and HOXB8 was determined by RIP assay. **f** Bioinformatics and luciferase reporter assays were respectively employed to conjecture and verify the binding site between miR-2682-5p and HOXB8. **g** RT-qPCR and western blot assays were performed to detect the mRNA expression and protein level of HOXB8. **h** Cells were transfected with pcDNA3.1, pcDNA3.1/ LINC01006, pcDNA3.1/LINC01006+miR-2682-5p, pcDNA3.1/LINC01006+sh-HOXB8#1; HOXB8 mRNA was measured in RT-qPCR. *P < 0.05, **P < 0.01, ***P < 0.001

Subsequently, we investigated the target gene of miR-2682-5p. With the employment of RNA22, microT and PicTar databases, HOXB8 was disclosed (Fig. 3a). HOXB8 expression in PC cell lines and normal human HPDE6-C7 was compared by RT-qPCR, and the results uncovered that HOXB8 was highly expressed in PC cell lines in comparison with HPDE6-C7 (Fig. 3b). The regulation of miR-2682-5p on HOXB8 expression was examined utilizing miR-2682-5p mimics and inhibitor. As shown in Fig. 3c, miR-2682-5p expression was efficiently knocked down by miR-2682-5p inhibitor in PANC-1 and BxPC-3 cells. Moreover, we found that HOXB8 expression was remarkably declined in PANC-1 and BxPC-3 cells with the addition of miR-2682-5p mimics, and remarkably lifted after treatment of miR-2682-5p inhibitor (Fig. 3d). RIP assay confirmed that LINC01006, miR-2682-5p and HOXB8 existed in RISC (Fig. 3e). We found that the 3′‐UTR of HOXB8 contains miR-2682-5p binding site. Luciferase reporter assays further verified that miR-2682-5p mimics remarkably decreased the luciferase activity of HOXB8‐WT reporter, but no obvious change of the luciferase activity of HOXB8‐Mut reporter was displayed between two groups (Fig. 3f). Later, RT‐qPCR certified the overexpression or knockdown efficiency of HOXB8 at mRNA and protein levels (Fig. 3g). Subsequently, rescue experiment elucidated that LINC01006 upregulation promoted the expression of HOXB8 in PANC-1 cells while HOXB8 knockdown or miR-2682-5p overexpression reversed the impact. Reciprocally, the depletion of HOXB8 caused by LINC01006 downregulation could be offset by either miR-2682-5p inhibition or HOXB8 overexpression in BxPC-3 cells (Fig. 3h). Briefly, HOXB8 is a target gene of miR-2682-5p in PC.

### LINC01006 promotes cell proliferation and migration in PC by sponging miR-2682-5p and modulating HOXB8 expression

**Fig. 4:**
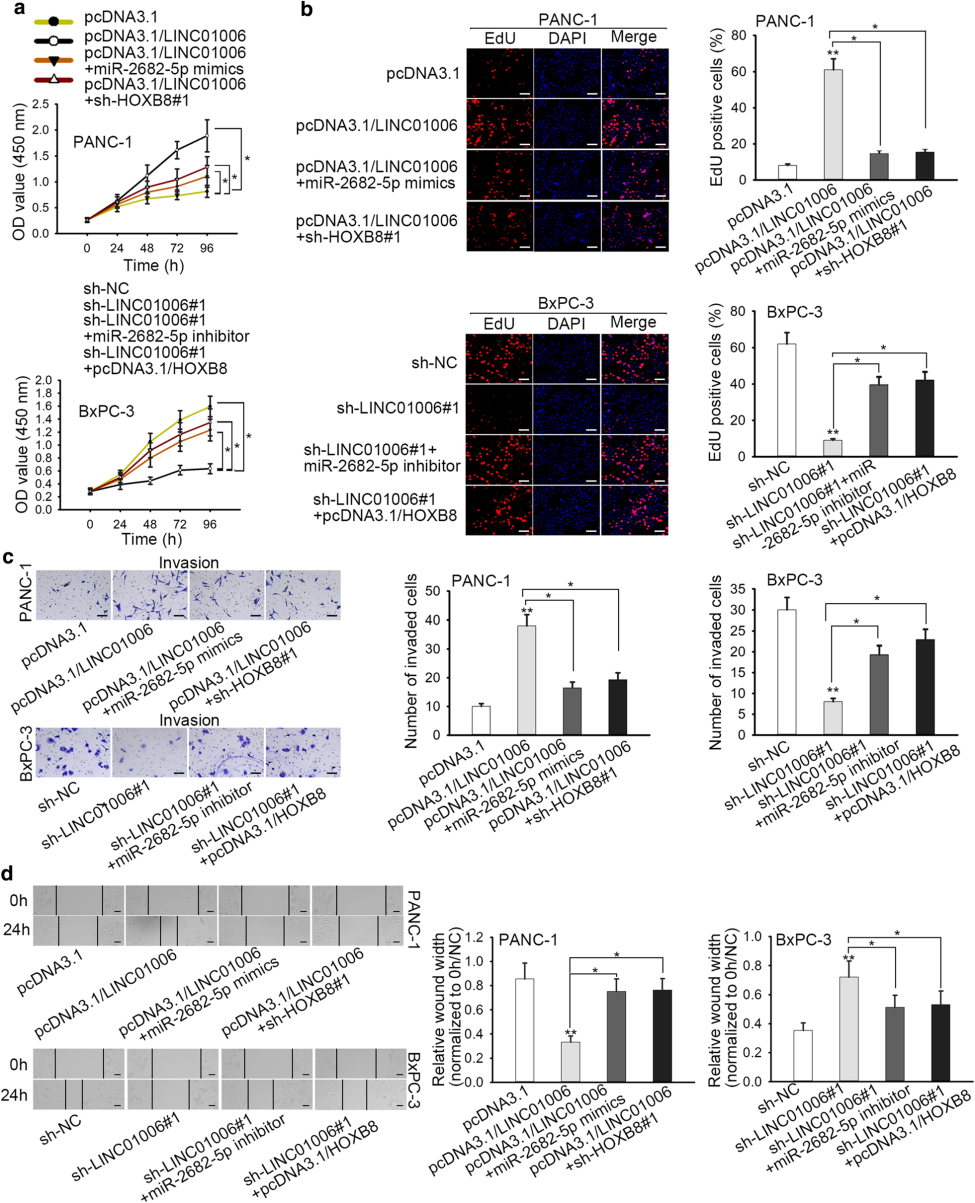
LINC01006 promotes cell proliferation and migration in PC by sponging miR-2682-5p and modulating HOXB8 expression. **a**, **b** CCK-8 and EdU assay were performed to examine the proliferation ability of transfected PC cells. **c** Transwell assay was exercised to check out cell invasion ability in different groups. **d** Wound healing assay was employed to test the migration ability of in PANC-1 and BxPC-3 cells transfected with different plasmids. *P < 0.05, **P < 0.01

To research the effects of LINC01006/miR-2682-5p/HOXB8 axis on PC progression, a few rescue assays were performed. CCK-8 and EdU assays revealed that LINC01006 overexpression‐mediated promotion on the proliferation of PANC-1 cells was rescued by miR-2682-5p mimics or HOXB8 knockdown, and LINC01006 knockdown‐induced inhibition on the proliferation of BxPC-3 cells was rescued by miR-2682-5p inhibitor or HOXB8 overexpression (Fig. 4a, b). Similarly, transwell assay elucidated that LINC01006 upregulation induced a marked increase of cell invasion in PANC-1 cells, and then overexpression of miR-2682-5p or silence of HOXB8 mitigated the changes caused by LINC01006 upregulation. Besides, LINC01006 knockdown induced a marked decrease of cell invasion in BxPC-3 cells, and then miR-2682-5p inhibition or HOXB8 knockdown abolished the changes induced by LINC01006 knockdown (Fig. 4c). Wound healing assay demonstrated that miR-2682-5p overexpression or HOXB8 depletion could reverse the effect of LINC01006 overexpression on cell migration in PANC-1 cells. And the effect of LINC01006 silencing on cell migration could also be countervailed by miR-2682-5p suppression or HOXB8 upregulation in BxPC-3 cells (Fig. 4d). In sum, LINC01006 promotes PC progression by sponging miR-2682-5p and modulating HOXB8 expression.

### HOXB8 acts as a transcription factor to modulate LINC01006 expression in PC

**Fig. 5:**
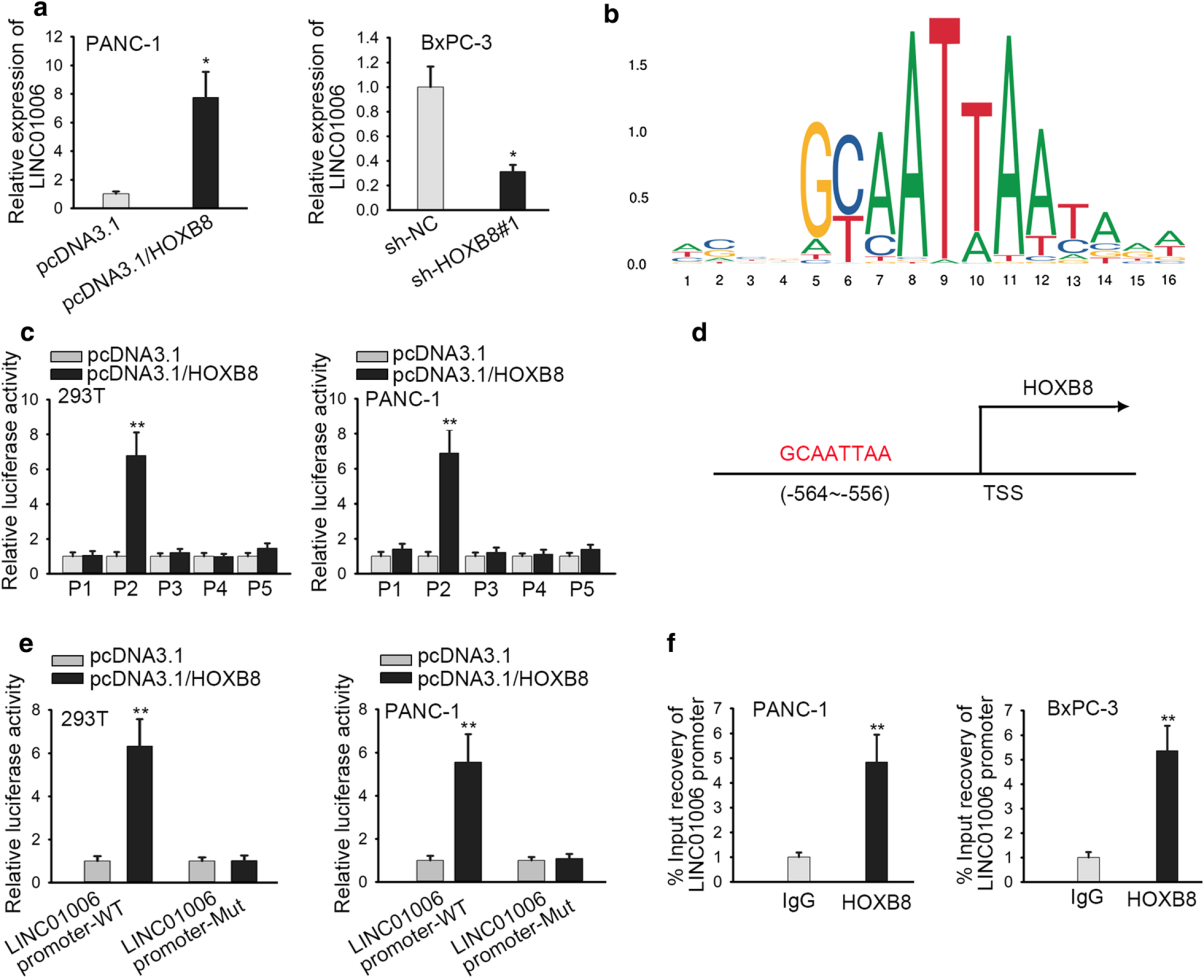
HOXB8 acts as a transcription factor to regulate LINC01006 expression in PC. **a** RT-qPCR assay was used to detect the overexpression and knockdown efficiency of HOXB8. **b** It exhibited the DNA motif of the transcription factor, HOXB8. **c** Luciferase reporter assay confirmed that HOXB8 activated the P2 promoter region activity. **d** Bioinformatics predicted the potential binding site of HOXB8 in LINC01006 promoter region. **e** Luciferase reporter assay confirmed that HOXB8 could bind with LINC01006 promoter. **f** ChIP assay verified the binding ability of HOXB8 in LINC01006 promoter region. *P < 0.05, **P < 0.01

It has been reported that HOXB8 serves as a transcription factor to promote the development of gastric cancer [24]. Herein we speculated its potential function on the regulation of LINC01006 in PC. Firstly, RT-qPCR results showed that LINC01006 expression could be markedly increased by HOXB8 upregulation in PANC-1 cells but decreased by sh-HOXB8#1 in BxPC-3 cells (Fig. 5a). Furthermore, the potential regulation of HOXB8 as a transcription factor on LINC01006 transcription was supported from JASPAR (http://jaspar.genereg.net/) predication. The DNA motif of HOXB8 was shown in Fig. 5b. The upstream 2000 bp of LINC01006 promoter has been divided into five parts including P1 (+ 1 to − 450), P2 (− 400 to − 850), P3 (− 800 to − 1250), P4 (− 1200 to − 1650), and P5 (− 1600 to − 2000). Luciferase reporter assay validated that the luciferase activity in P2 region was significantly strengthened after HOXB8 expression was upregulated (Fig. 5c). Bioinformatics analysis predicted that HOXB8 had a binding site (P2) in LINC01006 promoter region (Fig. 5d). Luciferase reporter assay further confirmed the wild LINC01006 promoter luciferase activity was promoted by HOXB8 upregulation but when P2 was mutated, the luciferase activity of LINC01006 promoter did not change upon HOXB8 upregulation (Fig. 5e). It was verified in ChIP assay that HOXB8 was abundant in LINC01006 promoter region (Fig. 5f). These results manifest that HOXB8 acts as a transcription factor to regulate LINC01006 in PC.

### The clinical expression of LINC01006/miR-2682-5p/HOXB8 axis in PC

From TCGA datasets, we disclosed that the expression of HOXB8 in PC tissues was not significantly upregulated compared with matched normal tissues while miR-2682-5p was promoted in PC tissues relative to normal ones (Additional file 3: Fig. S3A). Pearson correlation analysis detected the expression relation among LINC01006/miR-2682-5p/HOXB8. No significant relation was found among their expressions (Additional file 3: Fig. S3B). In addition, we also compared the level of LINC01006, miR-2682-5p and HOXB8 within PC tissues and normal ones. Results indicated the suppressed level of miR-2682-5p in PC tissues (n = 30) while the highly expressed level of LINC01006 and HOXB8 in PC tissues (n = 30) (Additional file 3: Fig. S3C). Regarding the expression correlation, correlation analysis pointed that LINC01006 expression was positively correlated with HOXB8 while inversely related to miR-2682-5p expression. Besides, it also exhibited a negative correlation between miR-2682-5p and HOXB8 (Additional file 3: Fig. S3D).

## Discussion

Aberrant expression of lncRNAs has been testified by existing literature to influence the malignant neoplasia in various cancers such as colorectal cancer, cholangiocarcinoma and liver cancer [15–17]. Despite the fact that LINC01006 has been figured out to participate in gastric cancer progression [21], the function of LINC01006 in PC remains to be studied. Our current work proved that LINC01006 was upregulated in PC tissues and cell lines, and LINC01006 silencing induced suppression in cell proliferation and metastasis in PC. In brief, LINC01006 is an oncogenic gene in PC.

More importantly, lncRNAs are discovered to serve as miRNAs sponges to influence the occurrence of a variety of cancers [25–27]. MicroRNAs (miRNAs), as another subtype of non-coding RNAs, also play crucial roles in the malignant process of cancers, having nucleotides ranging from 20 to 24 [28, 29]. As an illustration, lncRNA-RMRP sponges miR-206 to enhance cell growth in gastric cancer [30]. LncRNA XIST facilitates cell proliferation and metastasis in hepatocellular carcinoma via targeting miR-194-5p/MAPK1 axis [31]. LncRNA HOTAIR acts as a tumor promoter in esophageal cancer via sponging miR-148a [32]. Our study revealed that miR-2682-5p expression was downregulated in PC cells, and LINC01006 could bind with miR-2682-5p as well as inversely regulated miR-2682-5p expression. To conclude, LINC01006 functions as a sponge of miR-2682-5p in PC.

Apart from being sponged by lncRNAs, the molecular mechanism of miRNAs in cancers also includes regulating their downstream mRNAs [33, 34]. For instance, miR-9-5p suppresses TGFBR2 expression to accelerate the progression of non-small cell lung cancer [35]. MiR-223 regulates STMN1 expression to modulate cell growth and metastasis in gallbladder cancer [36]. MiR-24-3p promotes carcinogenesis of bladder cancer via targeting DEDD [37]. As an mRNA, homeobox B8 (HOXB8) has been validated to serve as a malignancy promoter in plenty of caners. For example, HOXB8 deficiency suppresses osteosarcoma progression through inactivating Wnt/β-catenin signaling pathway [38]. Absence of HOXB8 inhibits the development of colorectal cancer by inactivating Wnt/β-Catenin signaling pathway [39]. Our study verified that HOXB8 was overexpressed in PC cells, and HOXB8 could bind with miR-2682-5p as well as its expression was negatively modulated by miR-2682-5p. Furthermore, rescue assays indicated that LINC01006 functioned as a sponge of miR-2682-5p and regulated HOXB8 to promote cell growth and migration in PC. HOXB8 has also been proved to be a transcription factor in gastric cancer [24]. Our study manifested that HOXB8 could promote the transcription of LINC01006 through binding with its promoter region.

## Conclusion

In sum, a conclusion can be reached that LINC01006/miR-2682-5p/HOXB8 feedback loop promotes cell growth and metastasis in PC, shedding light on the further understanding of molecular mechanisms linked to PC occurrence.

## Supplementary information

**Additional file 1: Figure S1.** (A) LncRNA-sep unveiled ten elevated lncRNAs in PC tissues compared with corresponding non-tumor tissues. (B) Expression of four lncRNAs in PC cells lines and normal controls. *P < 0.05, **P < 0.01.

**Additional file 2: Figure S2.** (A) Images of tumors from sh-NC or sh-LINC01006 group. (B) The tumor growth curves of tumors in sh-NC group or sh-LINC01006 group. (C) Weight measurement of tumors in sh-NC group or sh-LINC01006 group. (D) Immumohistochemical staining of Ki-67 and PCNA in indicated tumors. (E)Immumohistochemical staining of lung metastasis nodes in two groups. **P < 0.01.

**Additional file 3: Figure S3.** (A) Expression profile of HOXB8 and miR-2682-5p from TCGA datasets. (B) Pearson correlation analysis of interrelation among LINC01006, HOXB8 and miR-2682-5p from TCGA datasets. (C) RT-qPCR assayed the expression of miR-2682-5p, HOXB8 and LINC01006 in PC tissues or matched normal samples. (D) The expression correlation among LINC01006, HOXB8 and miR-2682-5p was analyzed via Pearson correlation analysis. **P < 0.01, ***P < 0.001

## Data Availability

Not applicable.

## Abbreviations

PC: pancreatic cancer
lncRNAs: long non-coding RNAs
FBS: fetal bovine serum
shRNA: short hairpin RNA
CCK‐8: Cell Counting Kit‐8
PBS: phosphate buffer saline
PFA: paraformaldehyde
RISC: RNA-induced silencing complex
MiRNAs: MicroRNAs
HOXB8: homeobox B8
