# Supplementary figures and images for: LINC01006 promotes cell proliferation and metastasis in pancreatic cancer via miR-2682-5p/HOXB8 axis
Source: Cancer Cell Int. 2019 Dec 2;19:320. doi: 10.1186/s12935-019-1036-2 (PMC6889337; doi:10.1186/s12935-019-1036-2)

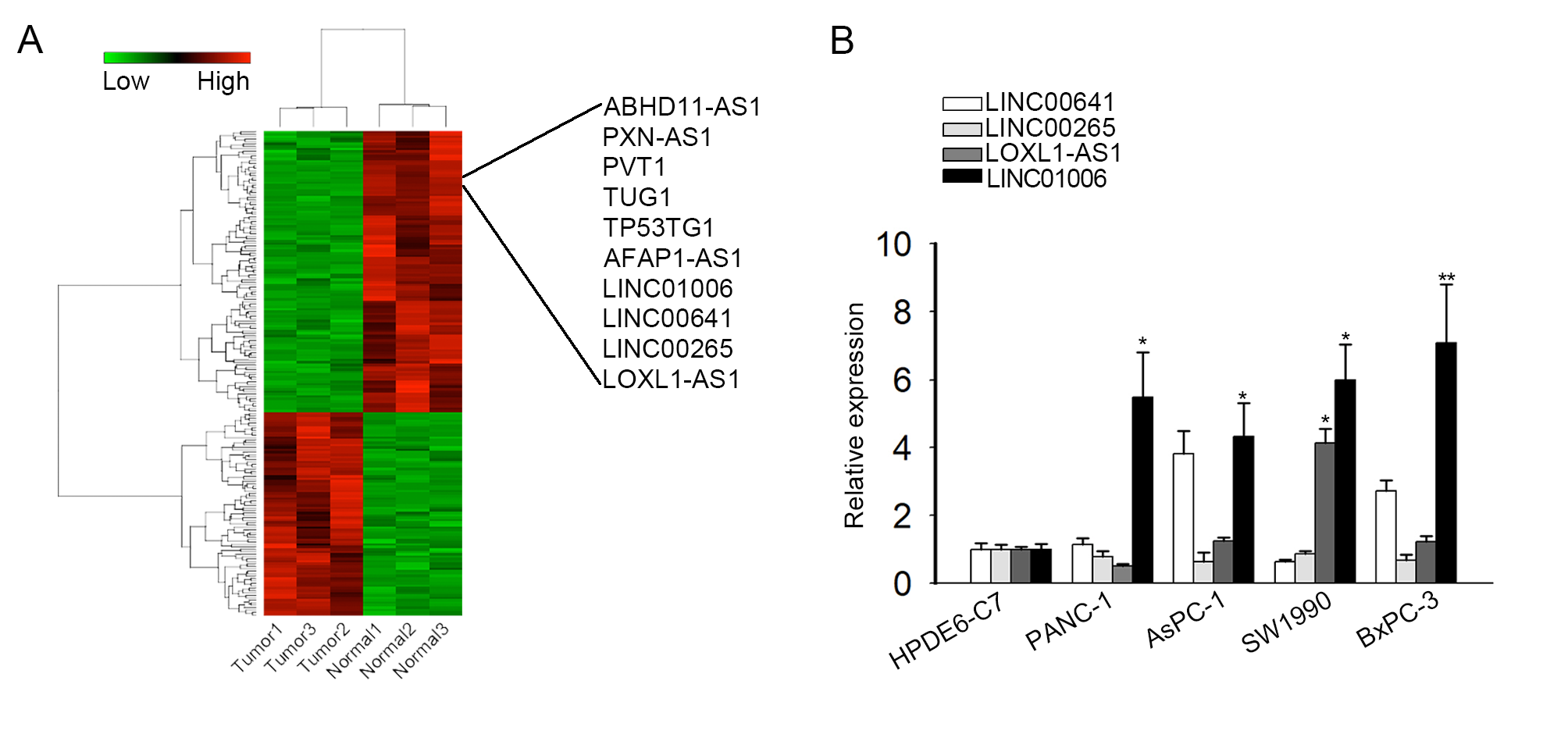

Supplement: Supplementary file 1 — Additional file 1: Figure S1. (A) LncRNA-sep unveiled ten elevated lncRNAs in PC tissues compared with corresponding non-tumor tissues. (B) Expression of four lncRNAs in PC cells lines and normal controls. *P < 0.05, **P < 0.01. [file 12935_2019_1036_MOESM1_ESM.tif]

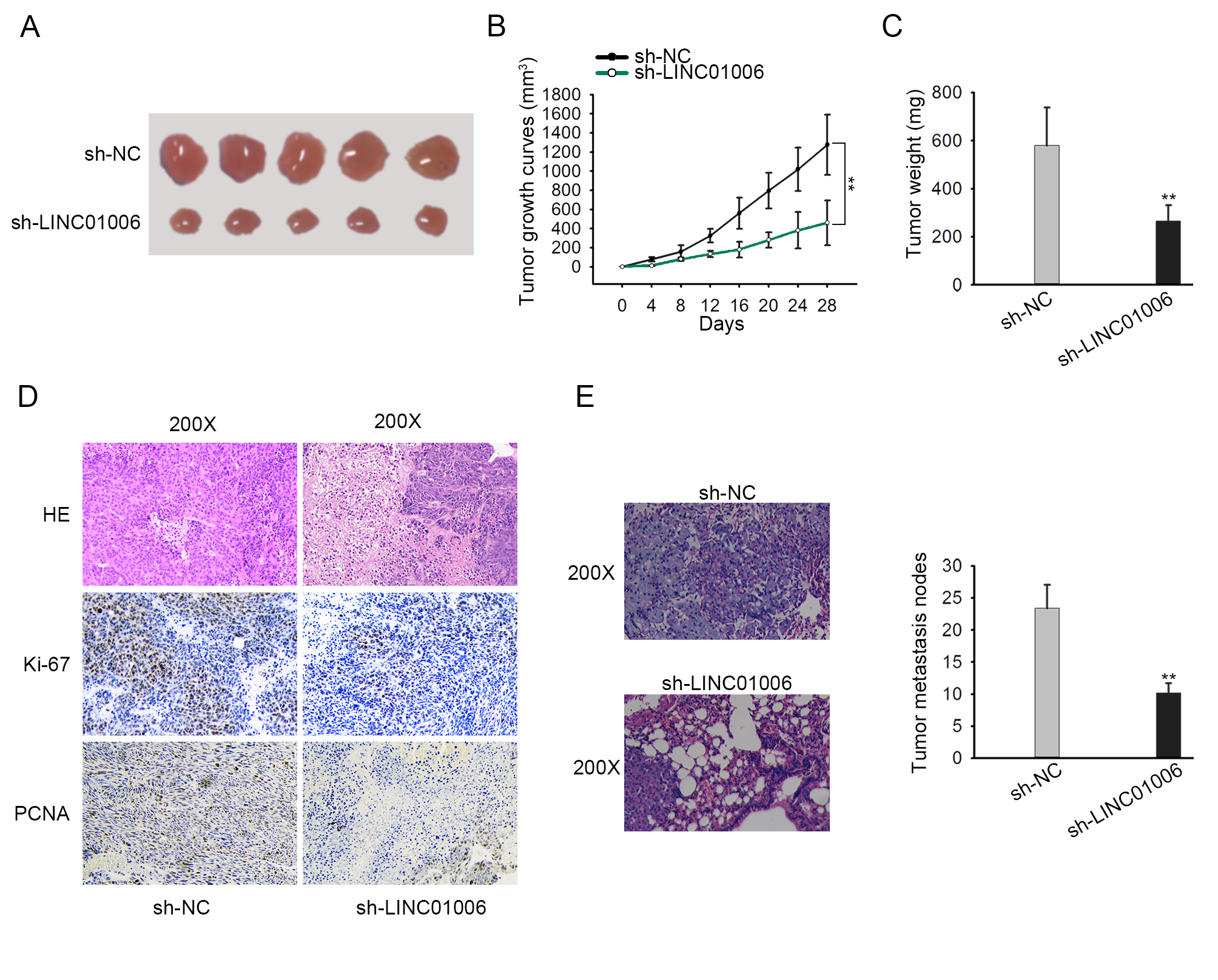

Supplement: Supplementary file 2 — Additional file 2: Figure S2. (A) Images of tumors from sh-NC or sh-LINC01006 group. (B) The tumor growth curves of tumors in sh-NC group or sh-LINC01006 group. (C) Weight measurement of tumors in sh-NC group or sh-LINC01006 group. (D) Immumohistochemical staining of Ki-67 and PCNA in indicated tumors. (E)Immumohistochemical staining of lung metastasis nodes in two groups. **P < 0.01. [file 12935_2019_1036_MOESM2_ESM.tif]

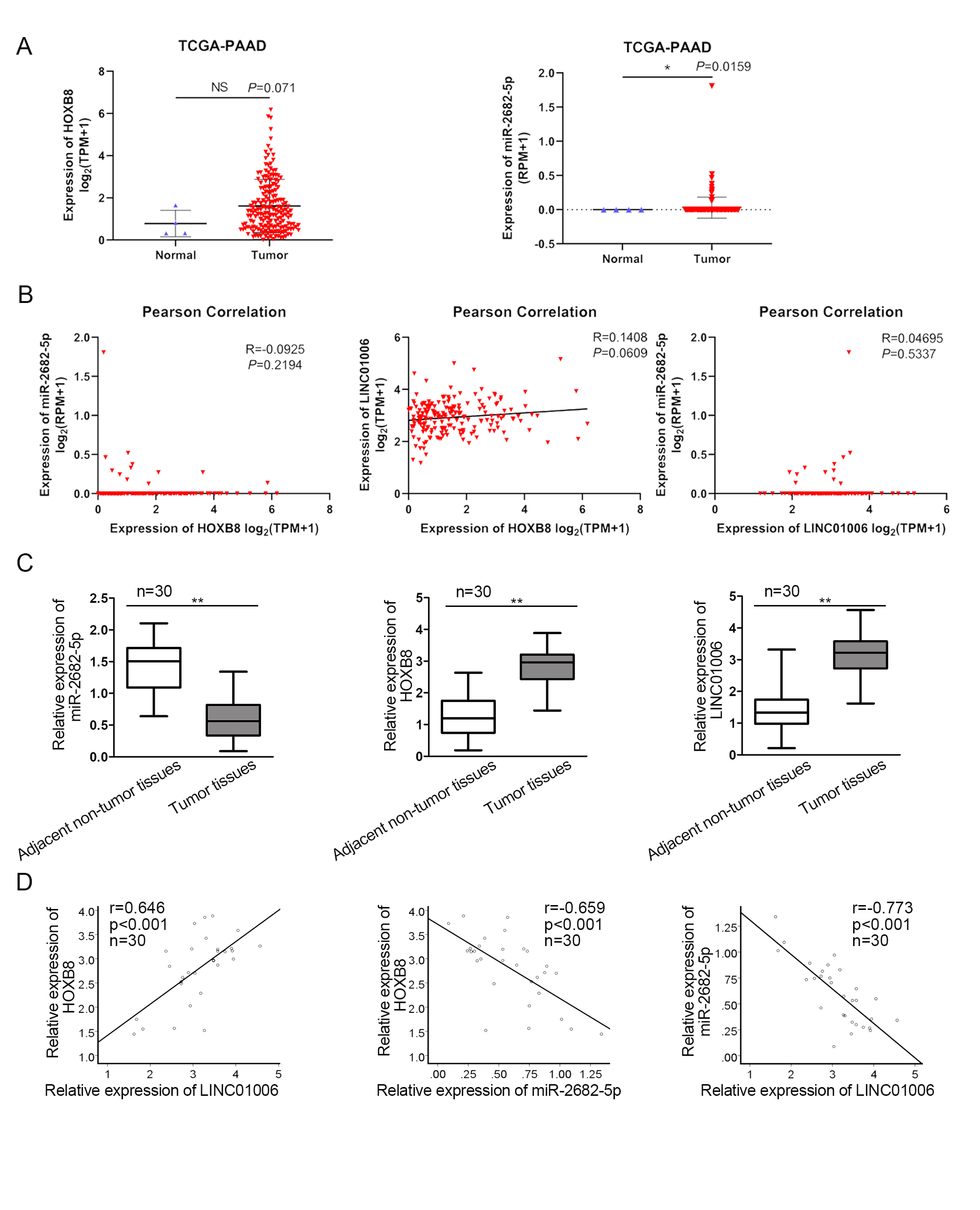

Supplement: Supplementary file 3 — Additional file 3: Figure S3. (A) Expression profile of HOXB8 and miR-2682-5p from TCGA datasets. (B) Pearson correlation analysis of interrelation among LINC01006, HOXB8 and miR-2682-5p from TCGA datasets. (C) RT-qPCR assayed the expression of miR-2682-5p, HOXB8 and LINC01006 in PC tissues or matched normal samples. (D) The expression correlation among LINC01006, HOXB8 and miR-2682-5p was analyzed via Pearson correlation analysis. **P < 0.01, ***P < 0.001 [file 12935_2019_1036_MOESM3_ESM.tif]
